# Reconstitution and characterization of eukaryotic N6-threonylcarbamoylation of tRNA using a minimal enzyme system

**DOI:** 10.1093/nar/gkt322

**Published:** 2013-04-25

**Authors:** Leo C. K. Wan, Daniel Y. L. Mao, Dante Neculai, Jonathan Strecker, David Chiovitti, Igor Kurinov, Gennadiy Poda, Neroshan Thevakumaran, Fang Yuan, Rachel K. Szilard, Elena Lissina, Corey Nislow, Amy A. Caudy, Daniel Durocher, Frank Sicheri

**Affiliations:** ^1^Samuel Lunenfeld Research Institute, Mount Sinai Hospital, Toronto, ON M5G 1X5, Canada, ^2^Department of Molecular Genetics, University of Toronto, Toronto, ON M5S 3E1, Canada, ^3^Cell Biology Program, Hospital for Sick Children, Toronto, ON M5G 1X8, Canada, ^4^Department of Chemistry and Chemical Biology, Cornell University, NE-CAT, Bldg. 436E, Advanced Photon Source, 9700 S. Cass Avenue, Argonne, IL 60439, USA, ^5^Medicinal Chemistry Platform, Ontario Institute for Cancer Research, MaRS Center, Toronto, ON M5G 0A3, Canada, ^6^Department of Biochemistry, University of Toronto, Toronto, ON M5G 3E1, Canada, ^7^Terrence Donnelly Centre for Cellular and Biomolecular Research, University of Toronto, 160 College Street, Toronto, ON M5S 3E1, Canada and ^8^Banting and Best Department of Medical Research, University of Toronto, 160 College Street, Toronto, ON M5S 3E1, Canada

## Abstract

The universally conserved Kae1/Qri7/YgjD and Sua5/YrdC protein families have been implicated in growth, telomere homeostasis, transcription and the N6-threonylcarbamoylation (t^6^A) of tRNA, an essential modification required for translational fidelity by the ribosome. In bacteria, YgjD orthologues operate in concert with the bacterial-specific proteins YeaZ and YjeE, whereas in archaeal and eukaryotic systems, Kae1 operates as part of a larger macromolecular assembly called KEOPS with Bud32, Cgi121, Gon7 and Pcc1 subunits. Qri7 orthologues function in the mitochondria and may represent the most primitive member of the Kae1/Qri7/YgjD protein family. In accordance with previous findings, we confirm that Qri7 complements Kae1 function and uncover that Qri7 complements the function of all KEOPS subunits in growth, t^6^A biosynthesis and, to a partial degree, telomere maintenance. These observations suggest that Kae1 provides a core essential function that other subunits within KEOPS have evolved to support. Consistent with this inference, Qri7 alone is sufficient for t^6^A biosynthesis with Sua5 *in vitro*. In addition, the 2.9 Å crystal structure of Qri7 reveals a simple homodimer arrangement that is supplanted by the heterodimerization of YgjD with YeaZ in bacteria and heterodimerization of Kae1 with Pcc1 in KEOPS. The partial complementation of telomere maintenance by Qri7 hints that KEOPS has evolved novel functions in higher organisms.

## INTRODUCTION

N6-threonylcarbamoyladenosine (t^6^A) is one of the 15 universally conserved tRNA modifications found in all three domains of life (1). With the sole exception of bacterial initiator tRNA^fMet^, t^6^A is present at position 37 of the anti-codon stem loop of all tRNAs with ANN recognizing anticodons (N being any nucleotide) (1). This modification serves to strengthen the codon–anticodon interaction and promote translational fidelity by the ribosome (2–4). In several subspecies of bacteria, fungi and plants, the t^6^A modification is processed further by dehydration into cyclic-t^6^A (5). Cells that are deficient for t^6^A modification exhibit an increased frequency of frameshift events and are prone to errors in AUG start codon selection, both of which can severely impair normal cellular physiology (6,7).

Comparative genomic analyses have implicated the Kae1/Qri7/YgjD and Sua5/YrdC protein families in the t^6^A biosynthesis pathway (8–10). In archaea and eukaryotes, Kae1 is part of an ancient macromolecular assembly known as KEOPS (kinase, putative endopeptidase and other proteins of small size). In yeast, KEOPS consists of five subunits, namely the Kae1, Bud32, Cgi121, Pcc1 and Gon7 proteins (11,12). Bud32 is one of the three ancient atypical protein kinases that are conserved in all archaeal and eukaryotic species, while Cgi121 and Pcc1 (a dimerization module) possess novel protein folds with no inferable functions (13). Gon7, to date only recognizable in fungi, remains the sole KEOPS subunit for which no structural information is available. In contrast to Kae1, YgjD orthologues function in a complex with two proteins, YeaZ and YjeE, both of which display no similarity in sequence to subunits within KEOPS.

Mutations within KEOPS in budding yeast gives rise to a set of diverse phenotypes including extreme slow growth, shortened telomeres and defective transcription coactivation by the SAGA and mediator complexes (11,12). The pleiotropic phenotypes of KEOPS have led to its initial classification as a telomere uncapping and elongation factor as well as a recruitment factor for transcriptional coactivators. Interestingly, Sua5/YrdC mutants are also defective in telomere length homeostasis in the budding yeast (14,15). As the posttranscriptional modification of tRNAs can influence seemingly unrelated cellular processes such as the DNA damage response and apoptosis (16–18) by modulating tRNA structure and function (19–21), it is possible that the pleiotropic effects of KEOPS and Sua5 mutations may be due entirely to defects in t^6^A metabolism, although this hypothesis has yet to be proven formally.

*In vitro* reconstitution studies have shed light on the mechanics of t^6^A formation*.* Reconstitution of t^6^A modification of tRNA was first achieved using TsaC/YrdC (the bacterial Sua5 orthologue), TsaD/YgjD (the bacterial Kae1 orthologue) and two bacterial-specific proteins TsaB/YeaZ and TsaE/YjeE (herein referred to as YrdC, YgjD, YeaZ and YjeE, respectively) (22) and most recently using archaeal/eukaryotic Sua5 and their corresponding KEOPS complexes (23). YrdC as an isolated protein was shown to generate threonylcarbamoyl-adenylate (TCA), a key early intermediate in the t^6^A reaction pathway (24). The specific direct or indirect roles that Kae1/YgjD and their known interacting partners play in the t^6^A synthetic process remains an enigma. To distinguish between direct and indirect contributions to t^6^A biosynthesis and to provide a framework to explore the accessory/regulatory role that an indirectly functioning subunit may play in t^6^A formation, we set out to identify and characterize a minimal eukaryotic t^6^A biosynthesis system. Toward this end, we show that yeast Sua5 and Qri7 (the mitochondrial paralogue of Kae1) are sufficient for t^6^A biosynthesis *in vitro*. Structural and functional characterization demonstrates that Qri7 forms dimers in solution, and that the integrity of the dimerization contact surface but not dimerization itself, is an essential component of its t^6^A biosynthetic function *in vitro* and biological function *in vivo*. Interestingly, the mode of dimerization is highly reminiscent of the Kae1–Pcc1 interaction in KEOPS (13) and the YgjD–YeaZ heterodimer (25) in the corresponding complex in bacteria. Together, this work provides new insight into how the universally conserved Kae1/Qri7/YgjD and Sua5/YrdC protein families function at a molecular level and provides a framework for probing how these evolutionarily conserved protein families impinge on multiple aspects of biology.

## MATERIALS AND METHODS

### Yeast growth rate analyses

The full collection of yeast strains used in this study is found in Supplementary Table 1. Yeast growth was monitored for 24 h by measuring OD_595_ every 15 min as described (26). Briefly, an overnight culture was diluted to an OD_600_ of 0.0625 with fresh medium (the *kae1Δ*, *bud32Δ*, *gon7Δ* and *cgi121Δ *strains were grown in SD-Leu; the *pcc1-4* strain was grown in SD-Leu-His) supplemented with 2% galactose. Growth assays were performed in a clear flat-bottom 96-well microplate (Greiner) sealed with adhesive plate seals (Cat. No. AB-0580, ABgene) using a custom-developed platform incorporating microplate reader TECAN GENios (Tecan-US, Durham NC, USA) and ACCESS software (26,27).

### Bulk tRNA isolation

Yeast strains were grown in 50 ml cultures to OD_600_ of 1.5 in their respective media as indicated above. Yeast strains were pelleted by centrifugation and resuspended in 500 μl of 10 mM Bis-Tris-HCl, pH 7.2, 10 mM EDTA and 0.5% sodium dodecyl sulphate. Total bulk RNA was extracted by adding one volume of 65°C phenol. Samples were incubated at 65°C for 30 min and shaken at 700 rpm. Total bulk RNA was isolated from the aqueous phase using a conventional phenol-chloroform extraction protocol. Isolation of bulk tRNA from total cellular RNA was performed using the Nucleobond® RNA/DNA 400 kit (Clontech Laboratories Inc.) according to the manufacturer’s protocol.

### Telomere length determination

Telomere length assays were performed as previously described (11).

### High performance liquid-chromatography analysis of tRNA composition

Isolated bulk tRNA or *in vitro* transcribed tRNA was digested and dephosphorylated according to previously described protocols (28). Ribonucleosides were analyzed on a Discovery C18 (15 cm × 4.6 mm, 5 μM) reverse-phase column (Supelco Analytical) equipped with an Ultrasphere ODS guard column (Beckman). Ribonucleosides were separated over a 35-min linear gradient of 98:2 (Buffers A and B, respectively) to 87.5:12.5 at a flow rate of 1.5 ml/min. The compositions of buffers A and B were 250 mM ammonium acetate, pH 6.5, and 40% acetonitrile, respectively. All High performance liquid-chromatography (HPLC) experiments were performed on a Dionex Ultimate 3000 HPLC Unit (Thermo Scientific) and data analysis was performed using the Chromeleon HPLC software.

### Recombinant Sua5 and Qri7 production

Yeast Sua5 (full length) and Qri7 (amino acids 30–407) were expressed in *Escherichia coli* BL21 (DE3) CodonPlus cells harboring an N-terminal hexa-histidine or glutathione-S-transferase tag. Proteins were purified using conventional affinity chromatography (Ni-NTA or glutathione sepharose) coupled to size exclusion chromatography in 25 mM HEPES, pH 7.5, 200 mM NaCl and 2 mM Dithiothreitol (DTT).

### *In vitro* tRNA transcription and purification

Overlap extension PCR was used to generate yeast tRNA^Ile^ and tRNA^Ala^ hepatitis delta virus ribozyme DNA fragments. The tRNA-ribozyme fragment were ligated into pUC19 and linearized with BamHI before runoff transcription reactions by T7 RNA Polymerase. Transcription reaction conditions were as follows: 100 mM Tris-HCl, pH 8.0, 4 mM ATP, 4 mM GTP, 4 mM CTP, 4 mM UTP, 10 mM DTT, 1 mM spermidine, 0.1% Triton X-100, 25 mM MgCl_2_, 30 μg/ml linearized DNA template, 0.2 mg/ml T7 RNA Polymerase, 10 U/ml thermostable inorganic phosphate (New England Biolabs) and 200 U/ml RiboLock™ RNase Inhibitor (Thermo Scientific). Transcription reactions were incubated for 4 h at 37°C and terminated by the addition of EDTA to a final concentration of 50 mM. RNA was isolated using a conventional phenol-chloroform extraction protocol. RNA pellets were solubilized in 8 M urea and refolded by rapid dilution into nine volumes of 25 mM Bis-Tris-HCl, pH 6.5, 1 mM MgCl_2_ and 0.2 mM EDTA, followed by overnight incubation at ambient temperature. The refolded tRNA was purified on a Source15Q column (GE Healthcare) using the following chromatography conditions: (i) four column volumes (CV) at 20% B and (ii) linear gradient over 25 CV from 20 to 30% B. Buffers A and B are 50 mM Bis-Tris-HCl, pH 6.5, 2 mM MgCl_2_ and 50 mM Bis-Tris-HCl, pH 6.5, 2 mM MgCl_2,_ 2 M NaCl, respectively. Fractions containing tRNA were pooled and further purified using size exclusion chromatography in buffer 20 mM Bis-Tris-HCl, pH 6.5, and 10 mM MgCl_2_.

### *In vitro* t^6^A reconstitution assay

t^6^A biosynthesis assays were performed in 50 mM Tris-HCl, pH 8.0, 200 mM NaCl, 1 mM DTT, 1 mM threonine, 1 mM NaHCO_3_, 2 mM ATP, 5 mM MnCl_2_, 2 μM of Sua5/Qri7 and 20 μM of tRNA. Reactions were incubated at ambient temperature for 60 min and stopped by the addition of EDTA to a final concentration of 25 mM. The tRNA was enzymatically digested and analyzed by HPLC as described above.

### Liquid chromatography–mass spectrometry analysis of small molecules

Instrumentation consisted of a U-HPLC system, HTS PAL autosampler and a 6538 Q-TOF mass spectrometer with an electrospray ionization source (Agilent Technologies). The reverse-phase ion pairing liquid chromatography method consisted of an Agilent RRHD Extend-C18 column (2.1 × 100 mm, 1.8 μm particle size, Agilent, Santa Clara, CA) and an aqueous/organic gradient run as described below. Mobile phase A consisted of 97:3 water:methanol with 10 mM tributylamine and 15 mM acetic acid. Mobile phase B consisted of methanol or acetonitrile as described below. For the analysis of t^6^A labeling (Supplementary Figure S3), the flow rate equaled 0.4 ml/min and the gradient using methanol as mobile phase B, extended from 0% B at 0.0 min, to 100% B at 5.5 min, to 0% B at 6.5 min, to 0% B at 7.5 min. For the analysis of TCA, the flow rate equaled 0.5 ml/min with methanol as mobile phase B for 0–23 min and acetonitrile supplied from a second pump as mobile phase B from 23–33 min. The gradient for the analysis of TCA was 1% B at 0 min, 1% B at 4 min, 20% B at 6.67 min, 35% B at 14.67 min, 35% B at 17.33 min, 60% B at 18.67 min, 60% B at 20 min, 90% B at 20.5 min, 99% B at 21.3 min, 99% B at 23.1 min, 1% B at 31 min, 1% B at 32 min. The column temperature was maintained at 37°C, and the autosampler at 4°C. The injection volume was 15 μl in a 10 μl loop. A lock mass mixture with ions at 112.9855 and 1033.9881 was continually supplied at 2 μl/min through a second nebulizer pressurized at 7 psig, and data were continuously recalibrated during the run to these masses. The quadrupole time of flight mass spectrometer was run in negative ionization mode with a data storage threshold of 50. Data were collected in high resolution mode at 2 Hz. Instrument run parameters were drying gas temperature 300°C, drying gas flow 10 L/min, sample nebulizer pressure 50 psig, ESI capillary voltage 3500 V, fragmentor voltage 115 V, skimmer 47 V, octopole 1 RF 750 V. The scan range was 25–1100 m/z. For MS/MS analysis of TCA, ions of m/z 491.1 and 495.1 were targeted using a collision energy of 15 (arbitrary units) using “medium” isolation of 4 m/z as specified by the manufacturer.

### Qri7 crystallization, structure determination and refinement

Qri7 (amino acids 30–407) intended for crystallography was purified as a 6×-HIS tagged protein. The 6×-HIS tag was cleaved using TEV protease and subsequently removed using a subtractive nickel purification. Qri7 was then purified in 20 mM HEPES, pH 7.5, 100 mM NaCl and 2 mM DTT using size exclusion chromatography with a Superdex 200 10/300 GL column (GE Healthcare). Crystals were grown at a protein concentration of 10 mg/ml in 0.1 M Tris-HCl, pH 7.5, 8% PEG8000 and 0.2 M MgCl_2_ by microseeding at 4°C. A seleno-methionine crystal data set was collected at the Advanced Photon Source NE-CAT beamline 24-ID-E using a constant wavelength of 0.97933 Angstroms at 93 Kelvin. The structure of Qri7 was solved by molecular replacement using PDB:2IVP as search model in PHASER (29), and refinement was carried out using BUSTER (30). The R_free_ value was calculated using 10% of the data set. The final refined structure showed Ramachandran statistics of 92.84, 5.73 and 1.43% in the preferred, allowed and outlier regions, respectively. The following residues were only modeled to Cβ due to a lack of side chain electron density: N60, L62, L65, K66, R96, N102, I107, I141, I152, D203, R207, K212, K222, Q226, T262, L264, Q277, R282, E310 and F348. The Qri7 coordinates were deposited to the PDB under the accession number 4K25.

### Analytical ultracentrifugation

All analytical ultracentrifugation experiments were performed on a XL-I unit (Beckman) at 20°C, and data analysis was performed using SEDFIT and SEDPHAT softwares (31). Sedimentation equilibrium experiments were performed at 0.25, 0.5 and 1 mg/ml protein concentrations at 10 000, 12 000, 15 000 rpm centrifuge speeds. The data were fitted against a monomer–dimer model for dissociation constant calculation. Sedimentation velocity experiments were performed at 0.3125, 0.0625, 0.125, 0.250, 0.5 or 1 mg/ml protein concentrations at speeds of 42 000 rpm at 8 h. Root mean square deviations (RMSD) for all sedimentation velocity data were <0.005.

### Refolding of his-Sua5

Purified recombinant 6xHIS-Sua5 was unfolded by dilution into 50 volumes of 50 mM HEPES, pH 7.5, 500 mM NaCl, 50 mM imidazole, 6 M guanidine-hydrochloride and further purified by nickel affinity chromatography. For refolding, the protein was concentrated and then rapidly diluted into 50 volumes of 25 mM HEPES, pH 7.5, 200 mM NaCl, 2 mM DTT and 50 mM arginine. The sample was then subjected to size exclusion chromatography for final purification.

### Small molecule docking of TCA into Qri7

Glide software (Schrödinger, Inc.) was used for the small molecule docking in this study. We first validated the performance of Glide against flexible molecules such as ATP and TCA [IUPAC name (2S,3R)-2-[[[(2R,3S,4R,5R)-5-(6-aminopurin-9-yl)-3,4-dihydroxyoxolan-2-yl]methoxy-hydroxyphosphoryl]oxycarbonylamino]-3-hydroxybutanoic acid)] that contain 10 and 12 freely rotatable bonds, respectively. To do so, we extracted the ligand (ATP) from the Kae1 crystal structure (PDB:2IVP) and docked it back into the apo-form of the protein. Before docking, geometries of the ligands were converted from 2D to 3D with LigPrep (Schrödinger, Inc.) using the OPLS2005 force field, and then the structures were ionized to pH 7.0 with Epik and energy minimized with default LigPrep settings. The receptor protein GRID was calculated for a large box that was defined in the presence of ATP at the binding site. A standard box size of 10 Å beyond each side of the ligand was used. Two H-bond constraints were defined that recognize the adenosine portion: the side-chain oxygen atom of Glu176 and the side-chain NH of Asn257, both of which are interactions observed in the 2IVP crystal structure. The hydroxyls of Thr12, Ser129 and Tyr280 located in the vicinity of the ligand were designated as rotatable groups. We used the default scaling of van der Waals radii of 0.8 for atoms with partial atomic changes <0.15. We generated 5000 poses during the docking run, and then selected the 400 best poses for postdocking minimization and saved the top five poses for each ligand. The best docking pose was close to that observed in the 2IVP structure (0.65 Å RMSD based on the all-atom superposition) and preserved all eight H-bonds between the protein and the ligand as well as coordination of the Fe^2+^ ion by the phosphate group of ATP.

This positive validation result encouraged us to proceed further and dock TCA into the refined structure of Qri7 following the identical procedure. The side chain of residue R218 was converted to an alternative rotamer to prevent steric hindrance to TCA. The resulting structure of TCA in Qri7 overlays closely with the adenosine, sugar and the first phosphate moieties of ATP in the 2IVP structure with Kae1.

### Small molecule docking of tobramycin into Qri7

The structure of TobZ bound to tobramycin (PDB:3VET) was superimposed onto the Qri7 structure in Coot by secondary structure matching (SSM superpose), which served to position tobramycin in the Qri7 catalytic cleft. Residues in Qri7 that made contact with the modeled TCA were determined using cCONTACT.

### Subcellular fractionation

The isolation of yeast mitochondria using subcellular fractionation was performed as previously described (32).

## RESULTS

### Bud32, Pcc1, Cgi121 and Gon7 play a supporting role to the essential function of Kae1/Qri7 in growth

In budding yeast, mutational analyses have shown that all five KEOPS subunits contribute to telomere homeostasis and t^6^A formation (8,9,11). Of the five KEOPS subunits, Kae1 is the sole subunit universally conserved across all three domains of life. Hence, we reasoned that Kae1 contributed an essential core function within KEOPS and that Bud32, Cgi121, Pcc1 and Gon7 evolved to augment or regulate Kae1. Interestingly, eukaryotic cells harbor a Kae1 paralog, Qri7, which is targeted to mitochondria (33). Consistent with the predicted paralogous relationship, redirection of Qri7 from the mitochondria to the cytoplasm via deletion of the mitochondrial targeting sequence enabled rescue of the slow growth phenotype of a *kae1Δ* yeast strain (34). Intriguingly, GFP-tagged Bud32, Gon7, Cgi121 and Pcc1 proteins do not localize to the mitochondria (33) and the yeast genome does not appear to encode mitochondria-targeted paralogues of Bud32, Gon7, Cgi121 and Pcc1. Taken together, these results raised the possibility that Qri7 and Kae1 share a common essential function, but that Qri7 operates in mitochondria in the absence of additional regulatory KEOPS subunits.

**Figure 1.**
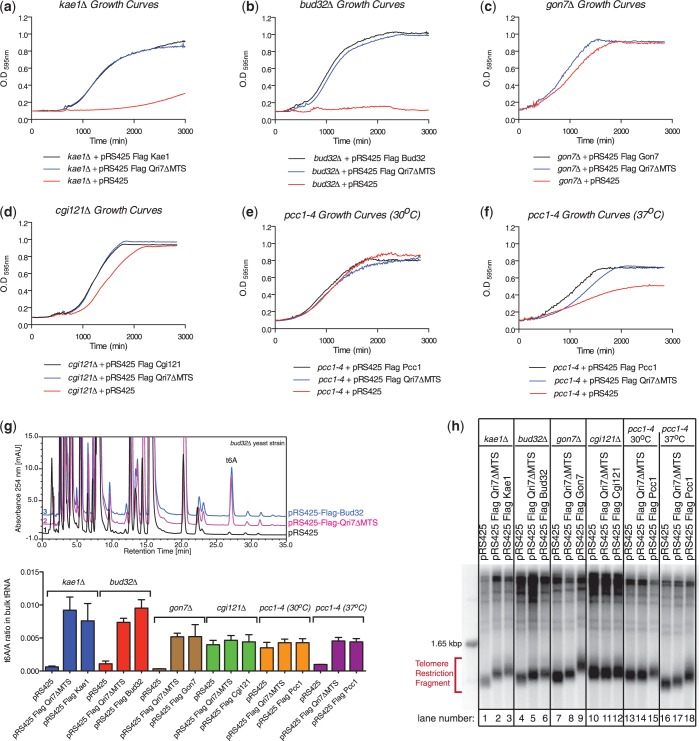
Qri7 functionally complements the slow growth, diminished t^6^A level and shortened telomere phenotypes of the KEOPS complex. A plasmid encoding Qri7 lacking its mitochondrial-targeting sequence (Qri7ΔMTS) was expressed in (**a**) *kae1*Δ, (**b**) *bud32*Δ, (**c**) *gon7*Δ, (**d**) *cgi121*Δ, (**e**) *pcc1-4* at the permissive temperature of 30°C and (**f**) *pcc1-4* at the nonpermissive temperature of 37°C. Expression of Qri7ΔMTS resulted in a full rescue of the slow growth phenotype in *kae1*Δ, *bud32*Δ, *gon7*Δ and *cgi121*Δ yeast strains and a partial rescue in the *pcc1-4* strain at its nonpermissive temperature. Plotted values represent mean values (*n* ≥ 4). (**g**) Representation of bulk tRNA nucleoside composition analysis by HPLC in *bud32*Δ yeast strains and the corresponding rescue experiments (top panel). Quantification of bulk tRNA t^6^A nucleoside level measurements normalized to the adenosine nucleoside levels in all null or mutated KEOPS yeast strains (bottom panel). Error bars represent standard deviation (s.d.) (*n* = 3). (**h**) Relative to the rescue observed for plasmid expression of the deleted or mutated gene, Qri7ΔMTS partially rescues the shortened telomere phenotype of *kae1*Δ̣(lanes 1–3), *bud32*Δ̣(lanes 4–6), *gon7*Δ̣(lanes 7–9), and *pcc1-4* at its 37°C nonpermissive temperature (lanes 16–18). *cgi121*Δ (lanes 10–12) does not display a significant telomere phenotype (compare lanes 10 and 12). The *pcc1-4* strain at its 30°C permissive temperature (lanes 12–15), displays a mild residual telomere phenotype. Within the resolution of the assay, this mild phenotype was not rescued by expression of Qri7ΔMTS.

If Bud32, Gon7, Cgi121 and Pcc1 play supporting roles only to Kae1’s essential core function, we reasoned that ectopic expression of Qri7 in the cytoplasm/nucleus should rescue not only the slow growth phenotype of a *kae1Δ* strain, but also the phenotypes of other KEOPS subunit deletions. We first confirmed that Qri7ΔMTS can rescue the *kae1Δ* slow growth phenotype and that this was due to a redirection of Qri7 protein from the mitochondria to the cytoplasm as predicted (Figure 1A and Supplementary Figure S1A). Expression of Qri7ΔMTS in a *bud32Δ* background rescued its associated slow growth phenotype (Figure 1B). Furthermore, the resulting growth rate was nearly identical to a *bud32Δ* strain harboring a Bud32 expression plasmid. Expression of Qri7ΔMTS in *gon7Δ* and *cgi121Δ* strains also resulted in growth rates identical to those observed for strains expressing Gon7 and Cgi121, respectively (Figure 1C and D). The temperature sensitive allele *pcc1-4* did not suffer a growth defect at the permissive temperature of 30°C (Figure 1E) but displayed a slow growth phenotype at the nonpermissive temperature of 37°C (12). At 37°C, expression of Qri7ΔMTS resulted in a partial rescue of the slow growth phenotype (Figure 1F). Consistent with Sua5 and Kae1/Qri7 proteins playing non-overlapping roles in the t^6^A biosynthetic process, Qri7ΔMTS did not rescue the slow growth phenotype associated with a *sua5Δ* strain (Supplementary Figure S1B). Together, these results indicate that Kae1/Qri7 provide the essential core function(s) of KEOPS that supports growth and are consistent with a model whereby Bud32, Cgi121, Gon7 and Pcc1 play supporting role(s) to the central essential function of Kae1.

### Bud32, Pcc1, Cgi121 and Gon7 play a supporting role to the essential function of Kae1/Qri7 in t^6^A biosynthesis and telomere length homeostasis

KEOPS mutant yeast strains additionally suffer from depletion of t^6^A modification and shortened telomeres (9,11). To determine if these phenotypes can be complemented by the expression of Qri7ΔMTS, we analyzed the t^6^A nucleoside levels in bulk tRNA extracted out of *kae1Δ*, *bud32Δ*, *gon7Δ, cgi121Δ* and *pcc1-4* yeast strains and those harboring the Qri7ΔMTS expression plasmid or the corresponding wild-type protein rescue plasmids (Figure 1G). We note that the t^6^A nucleoside we detect in our HPLC analyses represents a hydrolytic product of the expected natural cyclic t^6^A product that is generated during the tRNA hydrolysis reaction (5).

As expected, we observed low levels of t^6^A nucleoside in the bulk tRNA of *kae1Δ*, *bud32Δ*, *gon7Δ* and *pcc1-4* cells, with the latter grown at its nonpermissive temperature. We suspect that the detectable residual levels of t^6^A nucleosides may originate from mitochondrial tRNAs, which are not expected to be affected by KEOPS mutations. We did not detect a diminished level of t^6^A nucleoside in our *cgi121Δ* strain and this parallels the weaker growth and telomere phenotypes observed for this strain (13). In each instance where the levels of t^6^A is diminished, the ectopic expression of Qri7ΔMTS or the wild-type protein corresponding to the deleted (*bud32Δ*, *gon7Δ*) or mutated (*pcc1-4*) genes resulted in a rescue of normal t^6^A nucleoside levels (Figure 1G).

We next analyzed telomere length in each of our KEOPS mutant strains and their counterpart rescue strains (Figure 1H). The *kae1Δ* strain displayed a short telomere phenotype, which could be fully rescued by ectopic expression of Kae1 and partially rescued by ectopic expression of Qri7ΔMTS. We observed a similar trend in our analyses of the *bud32Δ* and *gon7Δ* strains. At the permissive temperature for *pcc1-4*, we observed a minor decrease in telomere length, likely due to residual effects of the temperature sensitive allele. Expression of wild-type Pcc1 at the permissive temperature resulted in a modest increase in telomere length. At the nonpermissive temperature, *pcc1-4* undergoes severe telomere shortening, which was rescued by ectopic expression of Pcc1 and partially rescued by ectopic expression of Qri7ΔMTS. The fact that Qri7 cannot fully reestablish telomere homeostasis hints that Kae1 and the larger KEOPS complex may have evolved novel functions that contribute to telomere maintenance in higher organisms.

Together, these results indicate that Qri7 provides an essential function(s) that supports t^6^A biosynthesis *in vivo* and, to a partial degree, telomere homeostasis. These results are consistent with a model whereby Bud32, Cgi121, Gon7 and Pcc1 play supporting role(s) to the central essential function of Kae1.

### Qri7 and Sua5 are both necessary and sufficient for t^6^A biosynthesis

**Figure 2.**
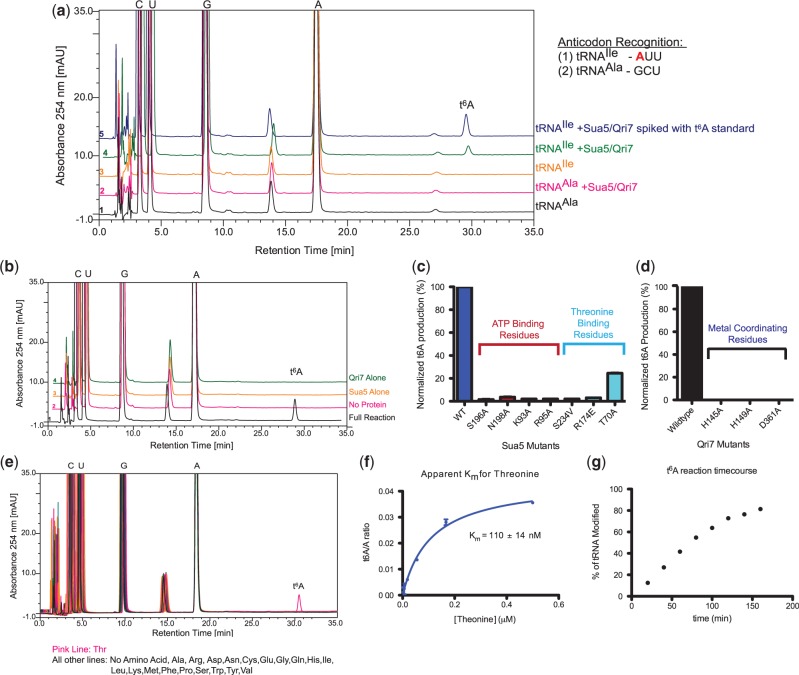
Sua5 and Qri7 are both necessary and sufficient for *in vitro* t^6^A biosynthesis. (**a**) HPLC nucleoside composition analysis of *in vitro* transcribed yeast tRNA^Ile^ (orange line) and yeast tRNA^Ala^ (black line). Incubation of yeast tRNA^Ile^ with Sua5/Qri7 (green line) in the presence of threonine, bicarbonate and ATP results in the formation of a novel nucleoside species while incubation of yeast tRNA^Ala^ with Sua5/Qri7 (pink line) does not result in the formation of any novel nucleoside species. Spiking the tRNA^Ile^ ribonucleoside sample with an internal t^6^A standard resulted in an increase in peak area for the novel nucleoside (blue line). The identities of the cytidine (C), guanosine (G), adenosine (A), uridine (U) and t^6^A peaks were confirmed by the addition of internal standards. (**b**) The formation of t^6^A *in vitro* requires both Sua5 and Qri7. HPLC nucleoside composition analysis of tRNA^Ile^ alone (pink line), tRNA^Ile^ incubated with both Sua5/Qri7 (black line), tRNA^Ile^ incubated with Qri7 (green line) and tRNA^Ile^ with Sua5 (orange line). (**c**) Mutation of Sua5 active site residues involved in ATP and threonine binding disrupts t^6^A biosynthesis. Error bars represent s.d. (*n* = 2) (**d**) Mutation of Qri7 active site residues involved in metal coordination completely abolishes t^6^A biosynthesis. (**e**) Sua5 and Qri7 specifically conjugate threonine onto tRNA^Ile^ (pink line) and not any of the other natural amino acids. (**f**) The dependency of t^6^A product formation versus threonine concentration for deriving an apparent Michaelis-Menten constant for threonine. Error bars represent s.d. (*n* = 2). (**g**) Time-course analysis of t^6^A modification by Sua5 and Qri7.

Based on the ability of Qri7 to complement the essential core function(s) of KEOPS in growth, t^6^A biosynthesis and telomere maintenance, we reasoned that Qri7 may lend itself to the reconstitution of eukaryotic t^6^A biosynthesis in the most simplistic manner. We thus tested yeast Sua5 and Qri7 for threonylcarbamoylation activity toward two *in vitro* transcribed yeast tRNAs. tRNA^Ile^ recognizes an ANN (specifically AUU) codon and hence is an expected target for t^6^A modification, whereas tRNA^Ala^ does not and hence should not be modified. We carried out *in vitro* reactions using Sua5, Qri7 and tRNA supplemented with threonine, bicarbonate and ATP, the known substrates for t^6^A formation (35,36). HPLC nucleoside composition analysis revealed a novel nucleoside species at the 30-min elution mark specifically for the tRNA^Ile^ reaction but not that containing tRNA^Ala^ (Figure 2A). Addition of a t^6^A internal standard resulted in an increase in peak area for the 30-min species, suggesting that the newly formed product was t^6^A. The identity of the novel peak was confirmed using mass spectrometry and by incorporation of heavy atom labeled ^13^C-threonine and ^13^C-bicarbonate (Supplementary Figure S3). In addition, the formation of t^6^A on tRNA^Ile^ required both Sua5 and Qri7 (Figure 2B). Notably, mutation of the conserved ATP and threonine-binding pockets of Sua5 [inferred from the structure of *Sulfolobus todokaii* Sua5; PDB 3AJE (37)] (Figure 2C and Supplementary Figure S2A) and the conserved active site metal-binding residues of Qri7 [inferred from the structure of *Pyrococcus abysii* Kae1; PDB 2IVP (38)] severely impaired t^6^A formation (Figure 2D and Supplementary Figure S2B). We conclude that the combination of Qri7 and Sua5 is necessary and sufficient for t^6^A biosynthesis.

In *E. coli* tRNA, only trace levels of amino acid cross-reactivity has been detected, namely the replacement of threonine with glycine to generate N6-glycylcarbamoyladenosine (39). This reflects the exquisite specificity of the t^6^A biosynthetic enzymes for threonine over all other amino acids. Consistent with the observed behavior *in vivo*, we can detect the formation of novel nucleoside products using Sua5 and Qri7 only upon the addition of threonine (Figure 2E) with an apparent Michaelis–Menten constant for threonine (K_m_^Thr^) of 110 ± 14 nM (Figure 2F). In our t^6^A reconstitution reactions, we typically observe >80% modification of input tRNA (Figure 2G).

In sum, we have reconstituted a threonine-specific eukaryotic t^6^A biosynthetic reaction using only Sua5 and Qri7 proteins. Our results indicate that the Sua5/YrdC and Kae1/Qri7/YgjD protein families form the essential core constituents of the t^6^A biosynthetic pathway and thus, the additional KEOPS subunits in archaea/eukaryotes, namely Bud32, Pcc1, Gon7, Cgi121 and additional protein factors in bacteria, namely YeaZ and YjeE, likely play indirect/regulatory roles in the t6A biosynthetic process.

### Reaction pathway analysis of the eukaryotic Sua5/Qri7 t^6^A biosynthesis system

**Figure 3.**
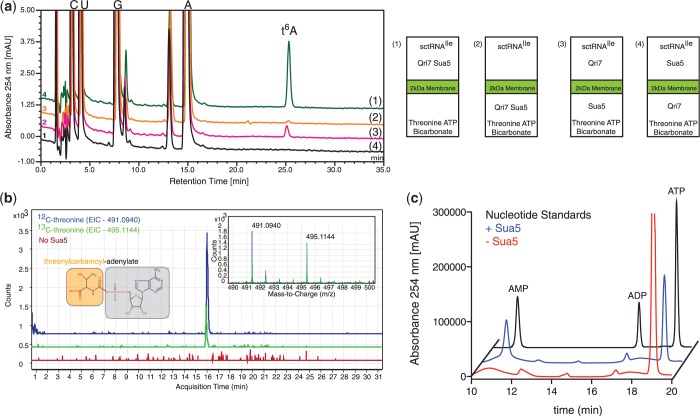
Sua5 functions upstream of Qri7 in the t^6^A biosynthetic pathway. (**a**) HPLC nucleoside composition analysis of tRNA^Ile^ that was incubated with Sua5/Qri7 in a chamber separated from threonine, bicarbonate and ATP by a 2 kDa membrane barrier (green line), which shows the formation of t^6^A. Separation of both Sua5/Qri7 from tRNA^Ile^ (orange line) does not give rise to t^6^A formation. Separation of Sua5 from Qri7 and tRNA^Ile^ (pink line) supports t^6^A formation while separation of Qri7 from Sua5 and tRNA^Ile^ does not support t^6^A formation (black line) (left panel). Cartoon representation of the experimental setup (right panel). (**b**) Incubation of Sua5 with threonine, bicarbonate and ATP results in the formation of TCA, which has a m/z value of 491.0940 (mass spectra inset—blue line). Reactions performed in the presence of ^13^C-labeled threonine resulted in a +4 shift in the m/z value to 495.1144 (mass spectra inset—green line). The extracted ion chromatograms (EIC) for both ^12^C and ^13^C-TCA are displayed by blue and green chromatograms respectively. Reactions preformed in the absence of Sua5 did not result in the formation and detection of TCA (mass spectra inlet – red line) and an extracted EIC at 491.0940 m/z is shown by a red chromatogram. (**c**) Ion-pair HPLC analysis of Sua5 ATP hydrolysis activity. An ATP, ADP and AMP standard mixture is shown (black line). In the presence of threonine and bicarbonate, Sua5 hydrolyzes ATP into AMP (blue line). In the absence of Sua5, ATP does not get hydrolyzed into AMP (red line).

To elucidate the reaction pathway of the eukaryotic Sua5/Qri7 t^6^A biosynthesis system, we used an experimental setup in which the t^6^A biosynthesis reaction was separated into adjacent reservoirs divided by a 2000 molecular weight cutoff membrane. We separated threonine, bicarbonate and ATP from Qri7, Sua5 and tRNA^Ile^ and were able to sustain t^6^A formation on tRNA^Ile^ (Figure 3A), which is consistent with the ability of threonine, bicarbonate and ATP to diffuse across the barrier membrane. Not unexpectedly, when both Sua5 and Qri7 enzymes were separated from tRNA^Ile^, the ultimate acceptor of t^6^A modification, t^6^A formation was abolished. When Sua5 was placed with threonine, bicarbonate and ATP in a reservoir separated from Qri7 and the tRNA, the production of t^6^A was still supported (Figure 3A). However, when Qri7 was placed with threonine, bicarbonate and ATP in one reservoir separated from Sua5 and tRNA, the production of t^6^A was abolished (Figure 3A). These experiments support the notion that Sua5 generates a diffusible reaction intermediate, and Qri7 in turn engages this product to carry forth the final conjugation step in t^6^A biosynthesis.

To identify the diffusible reaction intermediate shuttling from Sua5 to Qri7, we incubated Sua5 with threonine, bicarbonate and ATP and analyzed the reaction sample by mass spectrometry. We identified TCA (m/z ratio = 491.0940) (Figure 3B) as the likely intermediate, which is consistent with the earlier finding that bacterial YrdC generates TCA (24). Incubation with ^13^C-labeled threonine into the reaction conditions caused a +4 m/z shift for this species (Figure 3B), confirming the product was derived from threonine. The product could also be fragmented into an AMP adduct (data not shown) confirming ATP as a precursor. Furthermore, the production of TCA by Sua5 was coupled to the hydrolysis of ATP into AMP, as detected by ion-pair chromatography (Figure 3C). The use of a hexahistidine-tagged Sua5 construct that was purified under denaturating conditions coupled to refolding (Supplementary Figure S4A) showed identical specific activity in t^6^A biosynthesis and TCA production (Supplementary Figure S4B and C), confirming that Sua5 (and not a bacterial protein contaminant) is both necessary and sufficient for the initial step of t^6^A synthesis.

**Figure 4.**
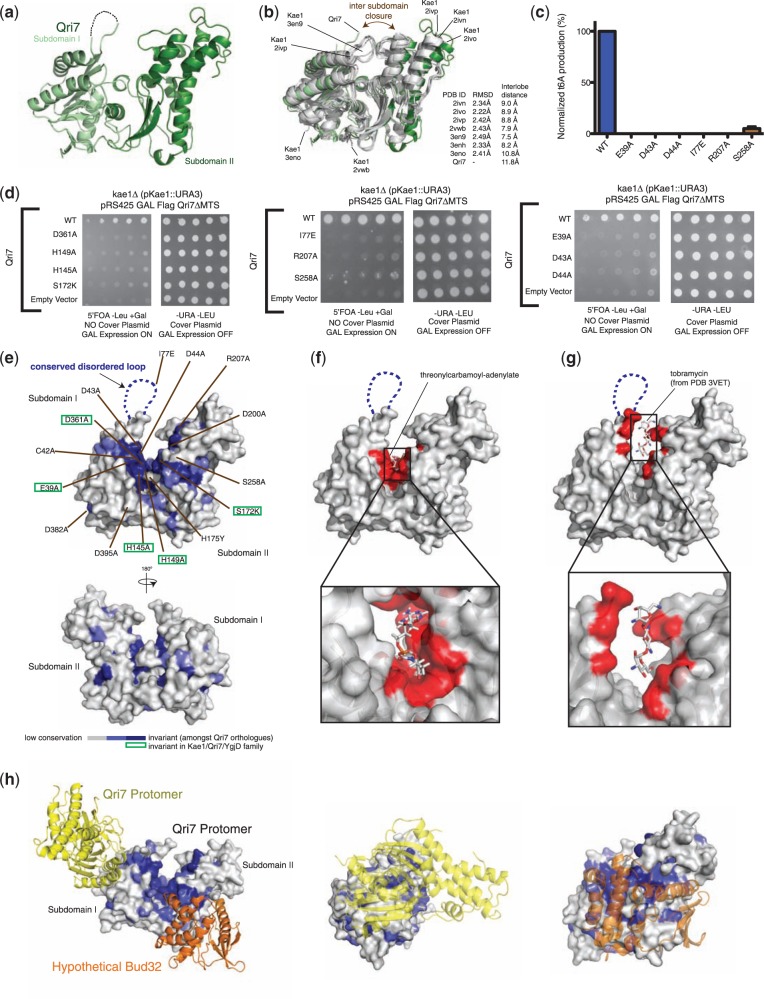
Structure–function analysis of Qri7. (**a**) Structure of *Saccharomyces cerevisiae* Qri7 (amino acids 30–407) shown in ribbons representation. Subdomains I and II are shown in light green and dark green, respectively. (**b**) Structural alignment of scQri7 (green) with all structures of Kae1 solved to date (grey), shown as ribbon representations. RMSDs of Qri7 relative to each Kae1 structure and the intersubdomain distances representing degree of lobe closure are listed. Intersubdomain distances were measured by the distance between Cα atoms of two conserved glycine residues (G117 and G174 in Qri7) on either side of the catalytic cleft. (**c**) Mutation of catalytic cleft residues on Qri7 abolishes t^6^A biosynthesis. Error bars represent s.d. (*n* = 2). (**d**) Mutation of catalytic cleft residues on Qri7 abolishes its ability to rescue the *kae1*Δ slow growth phenotype. (**e**) Projection of conservation onto a surface representation of Qri7. Residues in the catalytic cleft region are labeled and invariant residues across the Kae1/Qri7/YgjD family are boxed in green. (**f**) Docking of TCA into the catalytic cleft of Qri7. Qri7 catalytic cleft residues in close contact (<5 Å separation) with the docked TCA are shown in red. (g) Modeled tobramycin in the catalytic cleft of Qri7. Residues in close contact (<5 Å separation) with the modeled tobramycin molecule are shown in red. (**h**) Surface conservation of the Qri7 dimerization interface and hypothetical Bud32 binding site. The second Qri7 protomer in the dimer is shown in yellow ribbon and Bud32 is shown in orange ribbon (left panel). Head-on view of the Qri7 homodimer interface (middle panel) and head-on view of the hypothetical Bud32 binding site on Qri7 (right panel).

The proposed reaction mechanism predicts that Qri7 binds directly to TCA and tRNA as substrates. We speculated that the Qri7 infrastructure previously attributed to ATP binding (38) actually engages TCA. Indeed, we were able to dock threonylcarbomoyl-adenylate into the catalytic cleft using the nucleotide binding mode in the *Pyrococcus abyssi* Kae1-ATP complex (38), with only minor side-chain reorientations (see methods, Figure 4F).

In summary, the eukaryotic t^6^A biosynthesis system functions first through Sua5, which generates TCA using its adenylation function and then secondly through Qri7, which binds to TCA to conjugate the threonyl-carbamoyl moiety onto directly bound tRNA substrates. The activities of Sua5 and Qri7 are not necessarily physically coupled, and the Kae1/Qri7/YgjD protein family contains the required infrastructure for interacting with both TCA and tRNA.

### Crystal structure of Qri7

It is intriguing that Qri7 can support t^6^A biosynthesis as an isolated protein, while Kae1 and YgjD orthologues operate within larger macromolecular assemblies. To shed light onto the structural basis for the unique functional properties of Qri7, we solved the structure of yeast Qri7 (Table 1 and Figure 4A). At first glance, the structure of Qri7 is highly similar to previously reported structures of archaeal Kae1 (13,38,40) (PDB 2IVN, 2IVO, 2IVP, 2VWB, 3EN9, 3ENH and 3ENO) (2.34 Å, 2.22 Å, 2.42 Å, 2.43 Å, 2.49 Å, 2.33 Å and 2.41 Å C_α_ RMSDs, respectively) (Figure 4B). Qri7 exhibits the prototypical bilobal structure with its catalytic region sandwiched between subdomains I and II. Crystal structures of Kae1 orthologues (like ASKHA fold proteins in general) display variability in interlobe closure, and the Qri7 structure is notable in adopting the most open configuration reported to date for a Kae1 orthologue (Figure 4B).

**Table 1.** Data collection and refinement statistics

	scQri7
*Data collection*	
Space group	P2_1_2_1_2
Cell dimensions	
*a*, *b*, *c* (Å)	64.7, 131.2, 48.0
Resolution (Å)	20.6–2.90 (3.24–2.90)
*R*_sym_	0.059 (0.27)
*I*/σ*I*	23.4 (3.30)
Completeness (%)	99.9 (99.6)
Redundancy	1.82 (1.85)
*Refinement*	
Resolution (Å)	20.6–2.90
No. reflections	9434 (2357)
*R*_work_/*R*_free_	0.24/0.29
No. atoms	
Protein	2698
Ligand/ion	74
*B*-factors	77.9
R.m.s. deviations	
Bond lengths (Å)	0.008
Bond angles (°)	1.10

Structure was determined from a single crystal.

Values in parentheses are for highest-resolution shell.

R_sym _= Σ*_h_*Σ*_i_* | *I_h,i_* − *I_h_*| / Σ*_h_*Σ*_i_ I_h,i_*, where *I_h_* is the mean intensity of the *i* observations of symmetry related reflections of *h*. R = ∑ | *F*_obs _− *F*_calc_ | / ∑*F*_obs_, where *F*_obs _= *F*_P_ and *F*_calc_ is the calculated protein structure factor from the atomic model.

Projection of conservation onto the Qri7 structure identified a large contiguous surface emanating from the catalytic cleft (Figure 4E). Because Qri7, unlike Kae1 and YgjD, functions in the absence of intermolecular regulatory subunits, we reasoned that this surface conservation corresponds to the core catalytic infrastructure required for t^6^A biosynthesis by the Kae1/Qri7/YgjD family. A portion of the conserved surface was accounted for by the expected binding sites of TCA and phosphate coordinating metal ions (Figure 4F). Additional conservation was apparent on the lateral surface of Qri7 and at the tip of subdomains I and II, the former of which includes a highly conserved disordered loop (Figure 4E and Supplementary Figure S2B). The binding of acceptor substrate (tobramycin) in the evolutionarily related TobZ crystal structure (41) identifies these unaccounted for conserved surfaces at the tips of subdomains I and II as the possible tRNA substrate-binding surface (Figure 4G). Consistent with an essential role for these regions, the site-directed mutants I77E, R207A, S258A and D43A were not able to complement the slow growth phenotype of the *kae1Δ* strain and were also devoid of t^6^A biosynthesis activity *in vitro* (Figures 2D, 4C and D), comparable to mutants with impaired TCA or metal ion binding functions.

Interestingly, modeling of a hypothetical Bud32 [based on the Kae1–Bud32 complex PDB 3ENH (13)] interaction with Qri7 showed that Bud32 overlaps with a large portion of the conserved lateral surface of Qri7. As the lateral conserved surface is functionally required for t^6^A biosynthesis, this overlap may imply an inhibitory function for Bud32 on the core t^6^A biosynthetic activity of Kae1 (Figure 4H).

### Qri7 forms homodimers in solution

**Figure 5.**
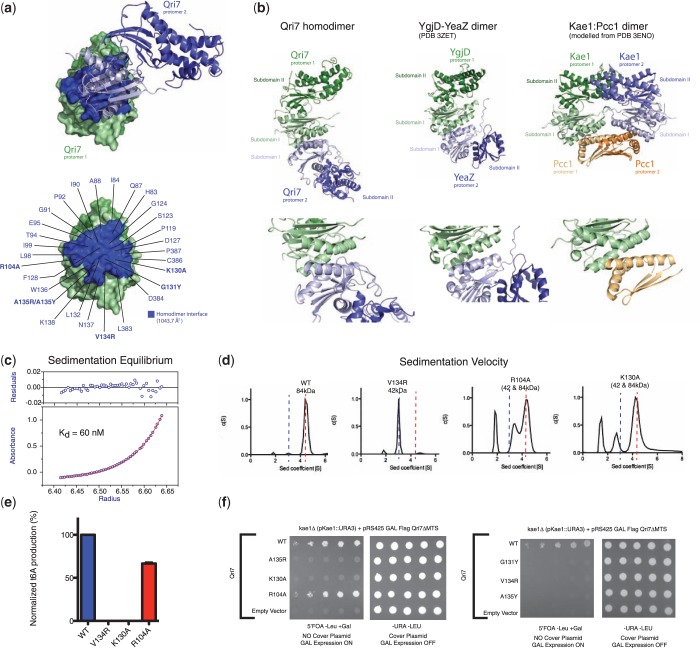
Qri7 forms homodimers in solution. (**a**) Two protomers of Qri7 in a homodimer configuration shown in surface and ribbons representation. On the surface represented protomer, the homodimerzation surface is highlighted in blue (top panel). Each residue at the dimerization interface is labeled and residues mutated for further characterization are shown in bold (bottom panel). (**b**) Ribbons representation and comparison of the homo and heterodimer configurations of Qri7:Qri7, YeaZ:YgjD and Kae1:Pcc1 complexes. Bottom panels display close up views of the dimer interfaces shown on top. (**c**) Sedimentation equilibrium analysis reveals a dimerization dissociation constant of 60 nM for Qri7. The red line denotes a fitted curve to a self-association model. The residuals for the fit are shown in the top panel. (**d**) Sedimentation velocity analysis of wild-type Qri7 (residues 30–407) and V134R, K130A and R104A mutants. Wild-type Qri7 behaves as a dimer in solution whereas mutant V134R behaves as a monomer. The K130A and R104A mutants exist in monomer-dimer equilibrium. Peaks with sedimentation coefficients <2 represent artifacts arising from small differences in buffer conditions between test and blank samples. (**e**) Dimerization mutants V134R and K130A are defective for t^6^A biosynthesis activity, whereas the R104A mutant retains partial activity. Error bars represent s.d. (*n* = 2). (**f**) Mutation of dimer interface residues abolishes the ability of Qri7 to rescue the slow growth phenotype of a *kae1*Δ yeast strain, with the exception of the R104A mutant.

Application of crystallographic symmetry to the Qri7 monomer structure revealed a dimer configuration with a large (2087.5 Å^2^) hydrophobic buried surface (Figure 5A and Supplementary Figure S2B), which is partially conserved across Qri7 orthologues (Figure 4H). Remarkably, the Qri7 dimerization surface is the same surface used by archaeal and eukaryotic Kae1 proteins to bind Pcc1, a dimerization module within KEOPS (13) (Figure 5B) and the same surface used by the bacterial orthologue YgjD to bind YeaZ (25) (Figure 5B). Interestingly, although the Pcc1 subunit in the Pcc1–Kae1 complex shares no recognizable sequence similarity to the Kae1, Qri7, YgjD or YeaZ proteins, its architecture resembles subdomain I (C-terminal lobe) of these proteins, and engages Kae1 through a four-helix bundle that is surprisingly similar to the Qri7 and YgjD-YeaZ modes of dimerization. This raises the intriguing question of whether Pcc1 evolved independently or from Kae1.

We confirmed that Qri7 forms homodimers in solution with a dissociation constant of 60 nM by equilibrium analytical ultracentrifugation (Figure 5C). To test whether Qri7 dimerization in solution is mediated by the same surface revealed by the crystal structure, we tested three individual interface mutants (R104A, K130A and V134R) for oligomerization status using velocity analytical ultracentrifugation. While wild-type Qri7 and five additional Qri7 mutant proteins harboring catalytic cleft mutations (D43A, D44A, I77E, R207A and S258A) existed exclusively as dimers (Supplementary Figure S5A), the V134R mutant existed exclusively as a monomer and the R104A and K130A mutants existed in monomer–dimer equilibrium (Figure 5D). The R104A and K130A mutants displayed a dimerization dissociation constant of 3.0 ± 0.3 μM and 2.5 ± 0.2 μM, respectively (Supplementary Figure S5B). These results confirm that Qri7 forms homodimers in solution through the same binding mode revealed in the crystal structure.

To determine whether dimerization is required for Qri7 function, we first tested the R104A, K130A and V134R mutants for t^6^A biosynthesis activity *in vitro*. The K130A and V134R mutants were devoid of t^6^A formation activity *in vitro*, whereas the R104A mutant displayed only a partial loss of enzymatic function (was 65% active) (Figure 5E). To determine if dimerization contributes to the ability of Qri7 to function *in vivo*, we tested the three above mutants as well as three additional dimer interface mutants for their ability to rescue the slow growth defect of the *kae1Δ* strain. Five out of the six mutations (K130A, G131Y, V134R, A135Y and A135R) abolished Qri7’s ability to rescue the slow growth phenotype of a *kae1Δ* yeast strain (Figure 5F). In contrast to the case for the K130A mutant, the finding that the R104A mutant was still able to rescue the growth of the *kae1Δ* yeast strain and partially sustain the t^6^A modification of tRNA, despite being partially compromised for dimerization function, suggests that the R104A mutation may impart a bypass for the necessity of dimerization. Together these studies highlight the importance of the dimerization contact surface but not dimerization *per se* for Qri7 function *in vitro* and *in vivo*.

The similarities in mode of dimerization observed for Qri7 and those observed for Kae1/YgjD orthologues that operate in larger complexes suggest that the potential to participate in protein–protein interactions using a common surface is a general requirement for t^6^A biosynthesis by the Kae1/Qri7/YgjD family. The ability for Qri7 to homodimerize using this surface provides a rationale for how it may be able to function in the absence of a Pcc1 dimerization module or a YeaZ protein–protein interaction and also how Qri7 can complement the essential function(s) of the entire KEOPS complex.

## DISCUSSION

### A universally conserved t^6^A biosynthetic pathway

Our reconstitution and functional studies demonstrate that Sua5 and Qri7 are both necessary and sufficient for the t^6^A modification of tRNAs. Whereas t^6^A formation by YgjD requires the additional presence of YeaZ and YjeE proteins and Kae1 requires the additional presence of the KEOPS complex (minimally *in vivo*), Qri7 appears to function in the mitochondria in the absence of a higher order assembly. The separation of Sua5 and Qri7 in their respective cytoplasmic and mitochondrial compartments suggests that t^6^A formation occurs via minimally two separable enzymatic steps and that Sua5 and Qri7 enzymes do not require physical coupling, a result supported by our barrier membrane studies. However, we cannot rule out that a small fraction of Sua5 protein resides within the mitochondria, which would eliminate the requirement for TCA, a highly unstable intermediate, to traverse the mitochondrial membrane.

**Figure 6.**
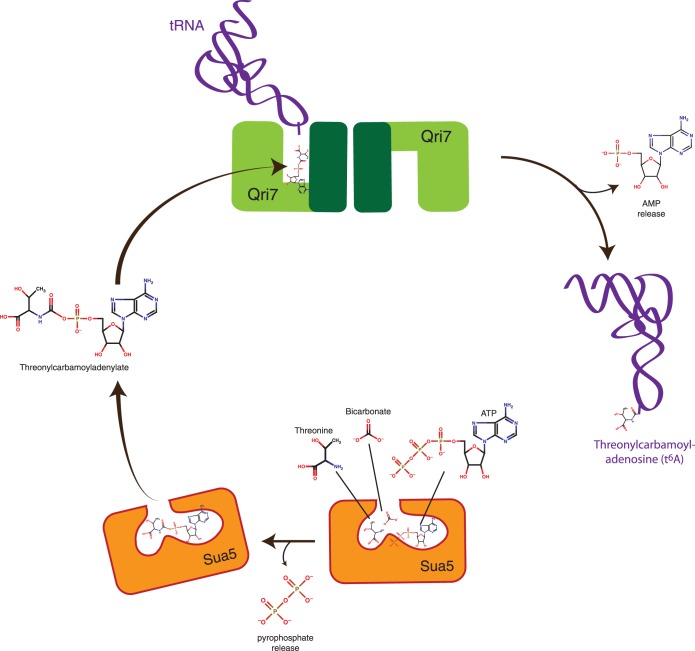
Minimal eukaryotic threonylcarbamoyladenosine (t^6^A) biosynthesis pathway using Sua5 and Qri7. Sua5 binds threonine, bicarbonate and ATP to generate TCA using its adenylation function. TCA is then released by Sua5 and bound to a Qri7 homodimer (modeled with one tRNA bound to its active site), which conjugates a threonylcarbamoyl-moiety onto bound tRNA substrates.

Our data supports the notion that Sua5 acts upstream in the t^6^A biosynthetic pathway whereby threonine, bicarbonate and ATP are converted to TCA (24) (Figure 6). TCA is released by Sua5 and then bound by Qri7, which can sample a homodimer configuration. Productive binding of the tRNA substrate positions adenosine 37 of the tRNA anticodon loop toward the active site of Qri7 to serve as an acceptor of threonylcarbamoylation (Figure 6). We reason that Qri7 binds to TCA using the nucleotide binding infrastructure of the ASKHA fold and does not require binding of additional ATP molecules to facilitate t6A biosynthesis. This would indicate that the Kae1/Qri7/YgjD family, representing one of the most ancient branches of the ASKHA family, is not an ATP hydrolyzing enzyme, which to our knowledge, is the only current example of a non-ATPase function for an ASKHA-fold protein.

Recent studies aimed at analyzing the bacterial t^6^A biosynthesis system have also discovered that YrdC functions upstream of the YgjD-YeaZ-YjeE complex (24). YrdC, the bacterial orthologue of Sua5, also generates TCA that feeds into the YgjD-YeaZ-YjeE complex. Together with our eukaryotic Sua5/Qri7 t^6^A biosynthesis system, this collection of findings suggests that t^6^A biosynthesis occurs through a universally conserved core pathway consisting of the YrdC/Sua5 and Kae1/Qri7/YgjD protein families. As the Kae1/Qri7/YgjD protein family diverged over the course of evolution, bacterial factors YeaZ and YjeE and archaeal/eukaryotic factors Bud32, Cgi121, Pcc1 and Gon7 may have evolved to play indirect roles in the t^6^A biosynthesis pathway, possibly to regulate the core enzymatic function of YgjD and Kae1, respectively.

### Functional relevance of the Kae1/Qri7/YgjD high-order assemblies

While the direct dimerization of Qri7 and the indirect dimerization of Kae1 by Pcc1 (13,42) appear important for protein function, the underlying basis for this requirement remains unclear. A possible function for dimerization may be to allosterically regulate intrinsic catalytic activity. This type of enzyme regulation is exemplified by the eIF2α, RAF and EGFR families of eukaryotic protein kinases. The kinase domains of all three families transition from monomers to specific dimer configurations (43–47), which are required for activation of protein kinase activity. Interestingly, a specific V600E mutation in B-RAF allows it to bypass dimerization as a prerequisite for function (48), a behavior reminiscent of the Qri7 R104A dimer interface mutant.

In the case of RAF and EGFR kinases, specific family members (KSR1 and KSR2 for RAF and Her3/ERBB3 for EGF) have evolved to solely serve as regulators by dispensing with catalytic function while maintaining their ability to dimerize. This phenomenon may account for the functional interplay within YgjD–YeaZ complexes where YeaZ (a structural orthologue of YgjD) has dispensed with catalytic function but still retains the ability to dimerize and hence regulate YgjD function. Interestingly, bacteria with reduced genomes such as *Mycobacterium genitalium* and *Candidatus Sulcia muelleri str. GWSS* do not encode YeaZ and YjeE proteins. It is tempting to speculate that YgjD in these minimal organisms have retained an ability to homodimerize and function in isolation, thus alleviating a necessity for YeaZ and YjeE-like proteins. If dimerization truly underpins the regulation of Qri7 and YgjD catalytic function, we reason that the binding of Pcc1 to the corresponding surface of Kae1 might serve a similar regulatory function. The atomic structure of Qri7 and the ability of Qri7 to participate in *in vitro* t^6^A biosynthesis as presented in this study provide a conceptual framework for future endeavors to uncover the precise molecular function of KEOPS regulatory subunits and their roles in t^6^A biosynthesis.

### The connection between tRNA biology and telomere maintenance

While Qri7 was able to fully rescue the growth defects and diminished t^6^A biosynthesis of KEOPS, it was only able to partially rescue the shortened telomere defect. This may reflect the fact that KEOPS has evolved new functions required for telomere maintenance that are not supplied by Qri7. One potential function may be the addition of new layers of regulation, which would allow for more precise temporal and spatial modulation of Kae1 enzymatic output. A second potential function could be the ability to recognize and modify novel RNA substrates, which presumably are not efficiently recognized by Qri7. Whatever the true basis for the incomplete ability of Qri7 to rescue the function of KEOPS in telomere maintenance, our findings provide a beachhead for future studies to decipher the precise role of KEOPS and the missing links between telomere and tRNA biology.

## SUPPLEMENTARY DATA

Supplementary Data are available at NAR Online: Supplementary Table 1 and Supplementary Figures 1–5.

Supplementary Data
