# Supplementary material for: Reconstitution and characterization of eukaryotic N6-threonylcarbamoylation of tRNA using a minimal enzyme system
Source: Nucleic Acids Res. 2013 Apr 25;41(12):6332–46. doi: 10.1093/nar/gkt322 (PMC3695523; doi:10.1093/nar/gkt322)
Supplement: Supplementary Data [file supp_41_12_6332__index.html]

Reconstitution and characterization of eukaryotic N6-threonylcarbamoylation of tRNA using a minimal enzyme system — Reconstitution and characterization of eukaryotic N6-threonylcarbamoylation of tRNA using a minimal enzyme system — Supplementary Data 

# Reconstitution and characterization of eukaryotic N6-threonylcarbamoylation of tRNA using a minimal enzyme system

## Supplementary Data

files

**Files in this Data Supplement:**

- Supplementary Data - pdf file
