# Supplementary material for: Reconstitution and characterization of eukaryotic N6-threonylcarbamoylation of tRNA using a minimal enzyme system
Source: Nucleic Acids Res. 2013 Apr 25;41(12):6332–46. doi: 10.1093/nar/gkt322 (PMC3695523; doi:10.1093/nar/gkt322)
Supplement: Supplementary Data [file supp_gkt322_nar-00014-r-2013-File003.pdf]

Reconstitution and characterization of eukaryotic N6- threonylcarbamoylation of tRNA using a minimal enzyme system

Leo C.K. Wan[1,2], Daniel Y.L. Mao[1], Dante Neculai[3], Jonathan Strecker[1,2], David Chiovitti[1], Igor Kurinov[4], Gennadiy Poda[5], Neroshan Thevakumaran[1,6], Fang Yuan[1,2], Rachel K. Szilard[1], Elena Lissina[2,7], Corey Nislow[2,7,8], Amy A. Caudy[2,7], Daniel Durocher[1,2,*], Frank Sicheri[1,2,6,*]

[1] Samuel Lunenfeld Research Institute, Mount Sinai Hospital, Toronto, ON M5G 1X5, Canada

[2] Department of Molecular Genetics, University of Toronto, Toronto, ON M5S 3E1, Canada

[3] Cell Biology Program, Hospital for Sick Children, Toronto, ON M5G 1X8, Canada

[4]Cornell University, Department of Chemistry and Chemical Biology, NE-CAT, Bldg. 436E, Advanced Photon Source, 9700 S. Cass Avenue, Argonne, IL 60439, USA

[5]Medicinal Chemistry Platform, Ontario Institute for Cancer Research, MaRS Center, Toronto, Ontario, Canada, M5G 0A3

[6]Department of Biochemistry, University of Toronto, Toronto, ON M5G 3E1, Canada

[7]Terrence Donnelly Centre for Cellular and Biomolecular Research, University of Toronto, 160 College Street, Toronto, ON M5S 3E1, Canada

[8]Banting and Best Department of Medical Research, University of Toronto, 160 College Street, Toronto, ON M5S 3E1, Canada

SUPPLEMENTARY DATA

*To whom c**orrespondence should be addressed**. Tel: +416-586-8471; Fax: +416-586-4800; Email: sicheri@lunenfeld.ca
Correspondence may also be addressed to D. Durocher. Tel: +416-586-4800x2544; Fax: +416-586-8869; Email: durocher@lunenfeld.ca

SUPPLEMENTARY TABLE AND FIGURES LEGENDS

Table S1. ***Saccharomyces cerevisiae*** **strains used in this study**

Supplementary Figure 1. Functional analysis of Qri7 and Sua5.
a) Analysis of Qri7 subcellular localization. C-terminal Flag tagged wild type Qri7 resides within the mitochondria (center panel). A C-terminal Flag tagged variant of Qri7 lacking its mitochondria targeting sequence (MTS) resides within the cytoplasm. Placement of a Flag tag adjacent to the MTS of wild type Qri7 also results in protein localization to the cytoplasm, as does further deletion of the MTS (right panel). WCE, C and M denote whole cell extract, cytoplasmic fraction and mitochondrial fraction respectively. PGK and Porin represent positive controls for cytoplasmic and mitochondrial localization (left panel). (b) Complementation analysis of a *sua5Δ* yeast strain by Qri7ΔMTS.

Supplementary Figure 2. Multiple sequence alignments of Sua5 and Qri7.
(a) Multiple sequence alignments of Sua5 generated by T-coffee. Organisms represented on alignment are *Escherichia coli* (ec), *Sulfolobus tokodaii* (st), *Danio rerio* (dr), *Thermoplasma acidophilum* (ta), *Caenorhabditis elegans* (ce), *Methanococcus jannaschii* (mj), *Homo sapiens* (hs), *Saccharomyces cerevisiae* (sc), and *Drosophila melanogaster* (dm). Identical residues are highlighted in dark orange and similar residues are highlighted by light orange. An asterisk denotes residues involved in ATP or threonine binding. Secondary structure elements shown at top of aligned sequences correspond to structure of *Sulfolobus tokodaii* Sua5; PDB 3AJE.
(b) Multiple sequence alignment of Qri7 generated by T-coffee. Organisms represented on alignments are *Ashbya gossypii* (ag), *Bos Taurus* (bt), *Drosophila melanogaster* (dm), *Homo sapiens* (hs), *Kluyveromyces lactis* (kl), and *Saccharomyces cerevisiae* (sc). Identical residues are highlighted in dark blue and similar residues are highlighted in light blue. Residues that are universally conserved in the Kae1/Qri7/YgjD family are highlighted in green. An asterisk denotes residues involved in Qri7 dimerization.

Supplementary Figure 3. Validation of t6A formation by mass spectrometry
(a) Mass spectra of t6A standard. (b) Mass spectra of *in vitro* generated t6A product, which shows a prominent m/z product of 411. (c) Mass spectra of *in vitro* generated t6A product in the presence of ^{13}C bicarbonate, which increased the abundance of t6A product with m/z value of 412 (+1). (d) Mass spectra of *in vitro* generated t6A product in the presence of ^{13}C threonine, which shifts the prominent t6A product to a m/z value of 415 (+4). (e) Mass spectra of *in vitro* generated t6A product with both ^{13}C bicarbonate and ^{13}C threonine, which shifts the prominent t6A product to a m/z value of 416 (+5). (f) Schematic of the atomic contribution of threonine and bicarbonate to t6A

Supplementary Figure 4. Refolded Sua5 has the same specific activity as a non-denatured preparation of Sua5
(a) SDS-PAGE analysis of his-Sua5 before and after refolding and re-purification (left panel). The additional low molecular weight bands in the his-Sua5 sample are results of Sua5 degradation during the denaturating and refolding process. Work flow chart of generating refolded his-Sua5 (right panel). (b) Refolded his-Sua5 retains the same

specific activity in t6A biosynthesis. (c) Refolded his-Sua5 retains the same specific activity in threonylcarbamoyl-adenylate production.

Supplementary Figure 5. Analytical ultracentrifugation analysis of Qri7 Mutants (a) Velocity analytical ultracentrifugation analysis of Qri7 proteins harboring catalytic cleft mutations. Mutations within the catalytic cleft do not disrupt the dimerization ability of Qri7. (b) The dimerization dissociation constant of R104A and K130A Qri7 dimer interface mutants. A protein concentration series for each mutant was analyzed by sedimentation velocity analytical ultracentrifugation. The peak size corresponding to the dimer species was normalized to the total peak size of both monomer-dimer species and plotted as a function of protein concentration.

Supplementary Table 1. *Saccharomyces cerevisiae* strains used in this study

Strain	Alias	Strain Background	Genotype	Source
DD2067	*bud32Δ*	W303	*MATa ade2-1 trp1-1 can1-100 leu2-3,112 his3-11,15 ura3-1 GAL+ psi+ ssd1-d2 RAD5+ bud32::NATMX*	Reference 11
DD2068	*kae1Δ*	W303	*MATα ade2-1 trp1-1 can1-100 leu2-3,112 his3-11,15 ura3-1 GAL+ psi+ ssd1-d2 RAD5+ kae1::KANMX*	Reference 11
DD572	*cgi121Δ*	W303	*MATa ade2-1 trp1-1 can1-100 leu2-3,112 his3-11,15 ura3-1 GAL+ psi+ ssd1-d2 RAD5+ cgi121::KANMX*	Reference 11
DD1239	*gon7Δ*	S288c	*MATa his3-1 leu2-0 met15-0 ura3-0 gon7::KANMX*	Reference 11
DD1872	*pcc1-4*	W303	*MATa ade2-1 trp1-1 can1-100 leu2-3,112 his3-11,15 ura3-1 GAL+ psi+ ssd1-d2 pcc1-4::HIS3, ade8D*	Reference 12
	sua5Δ	S288c	*MATα his3-1 leu2-0 ura3-0 sua5::KANMX*	See Acknowledgement

a

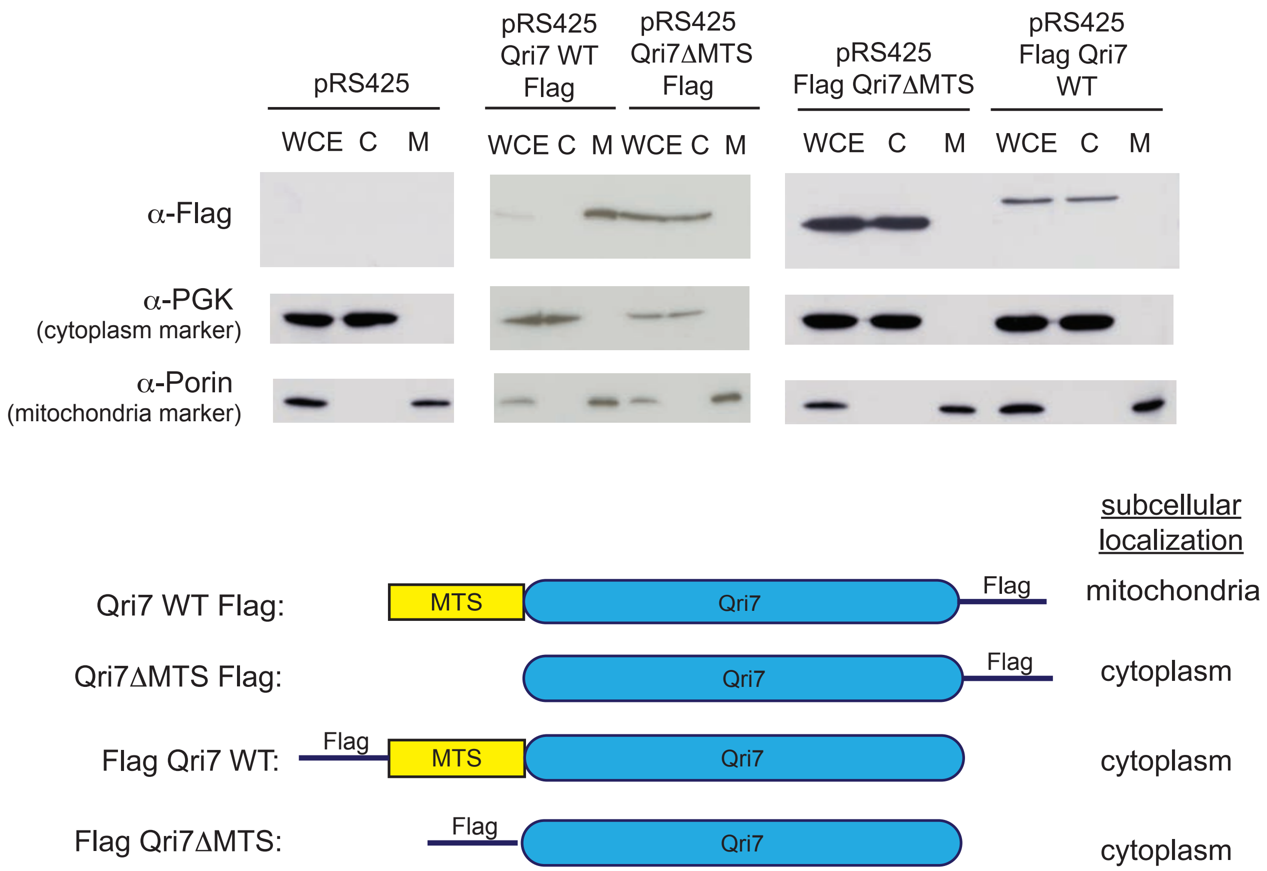

b

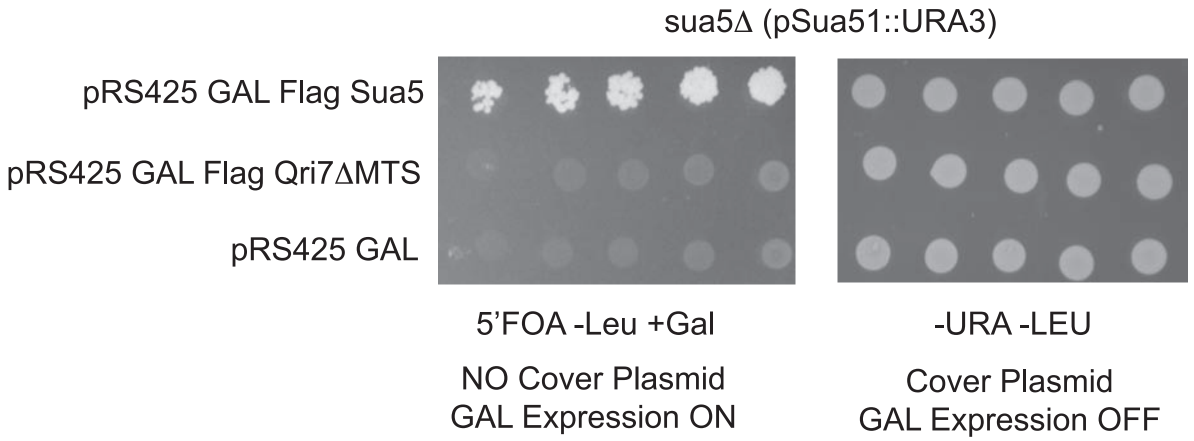

Supplementary Figure 1

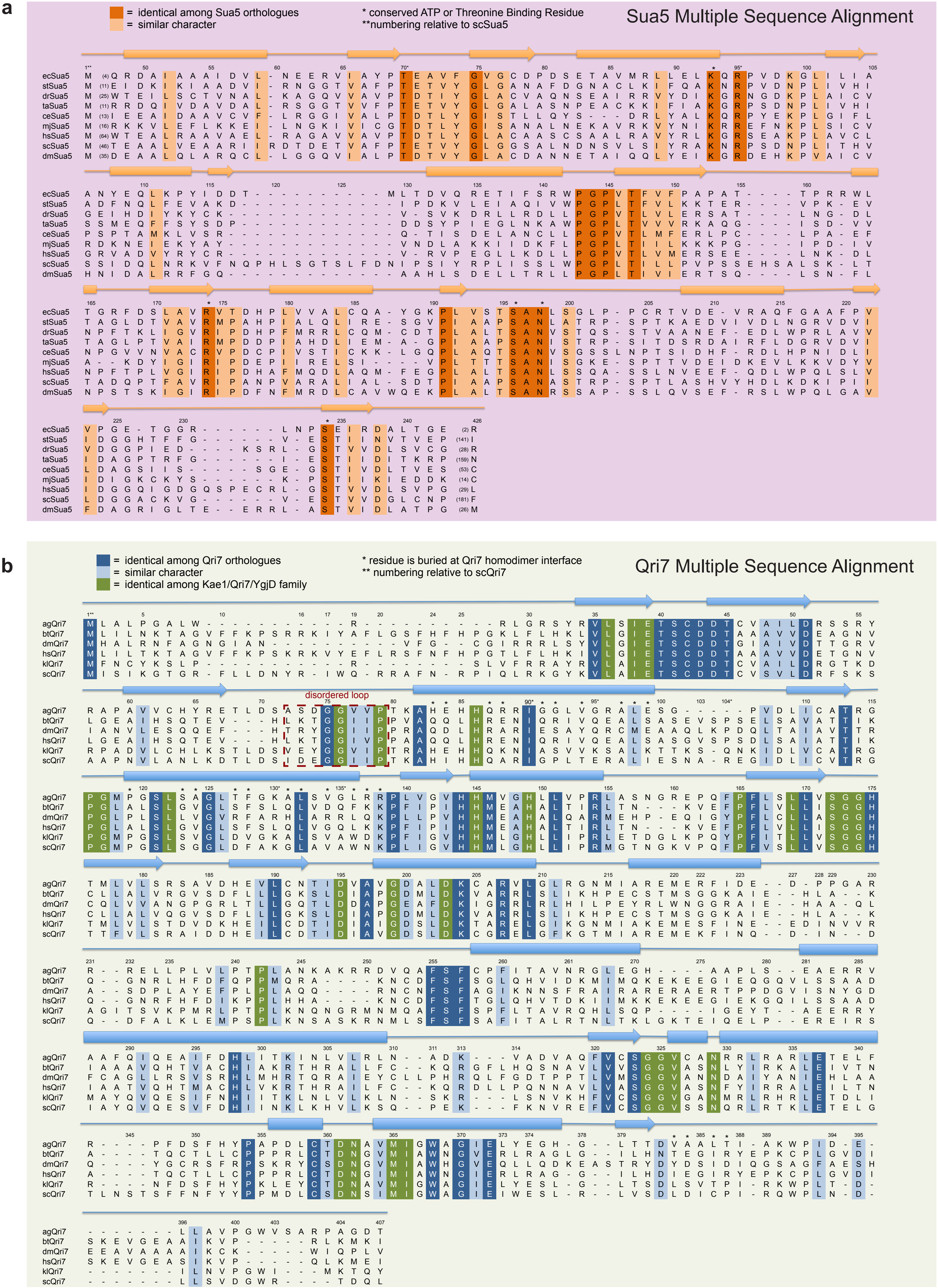

Supplementary Figure 2

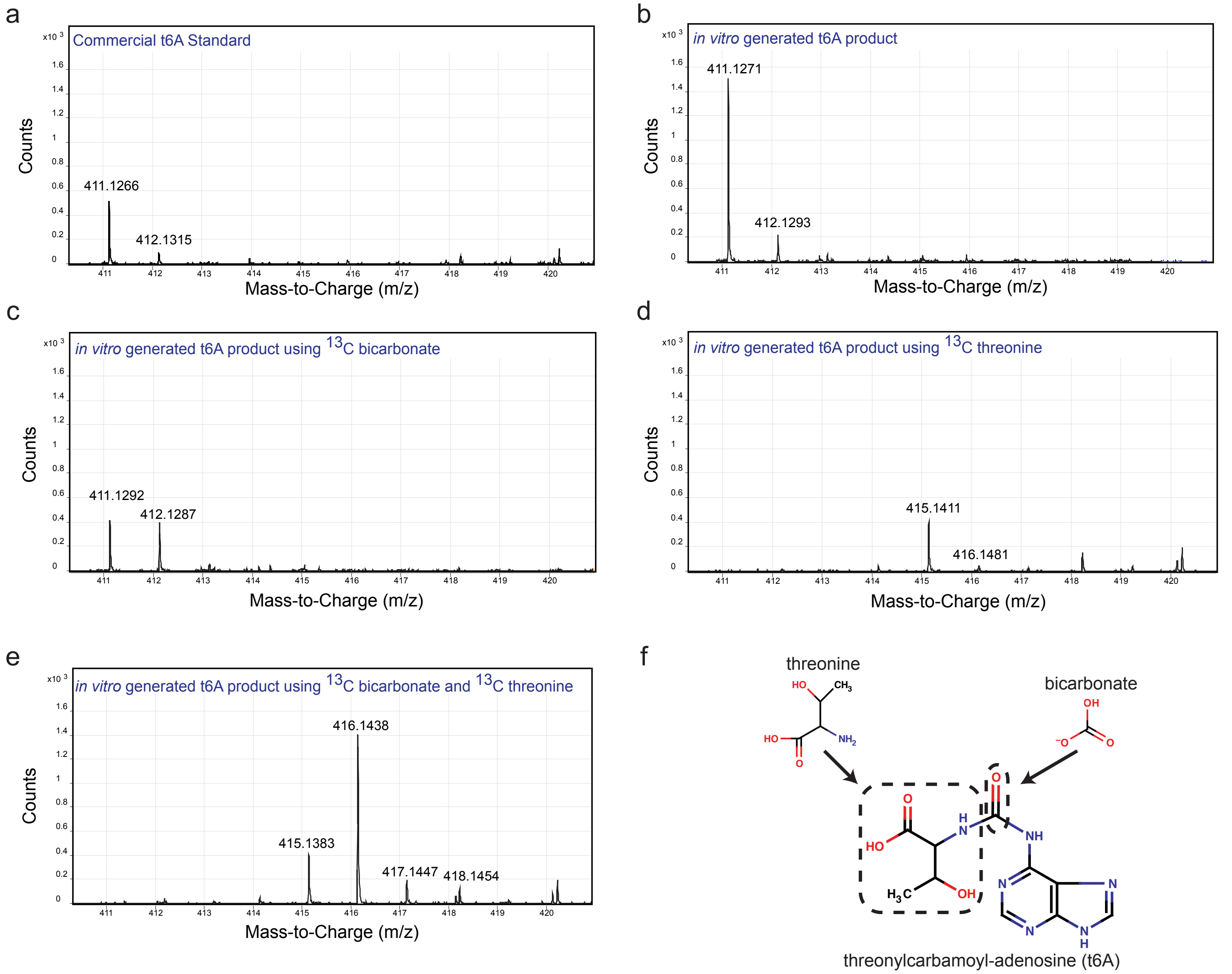

Supplementary Figure 3

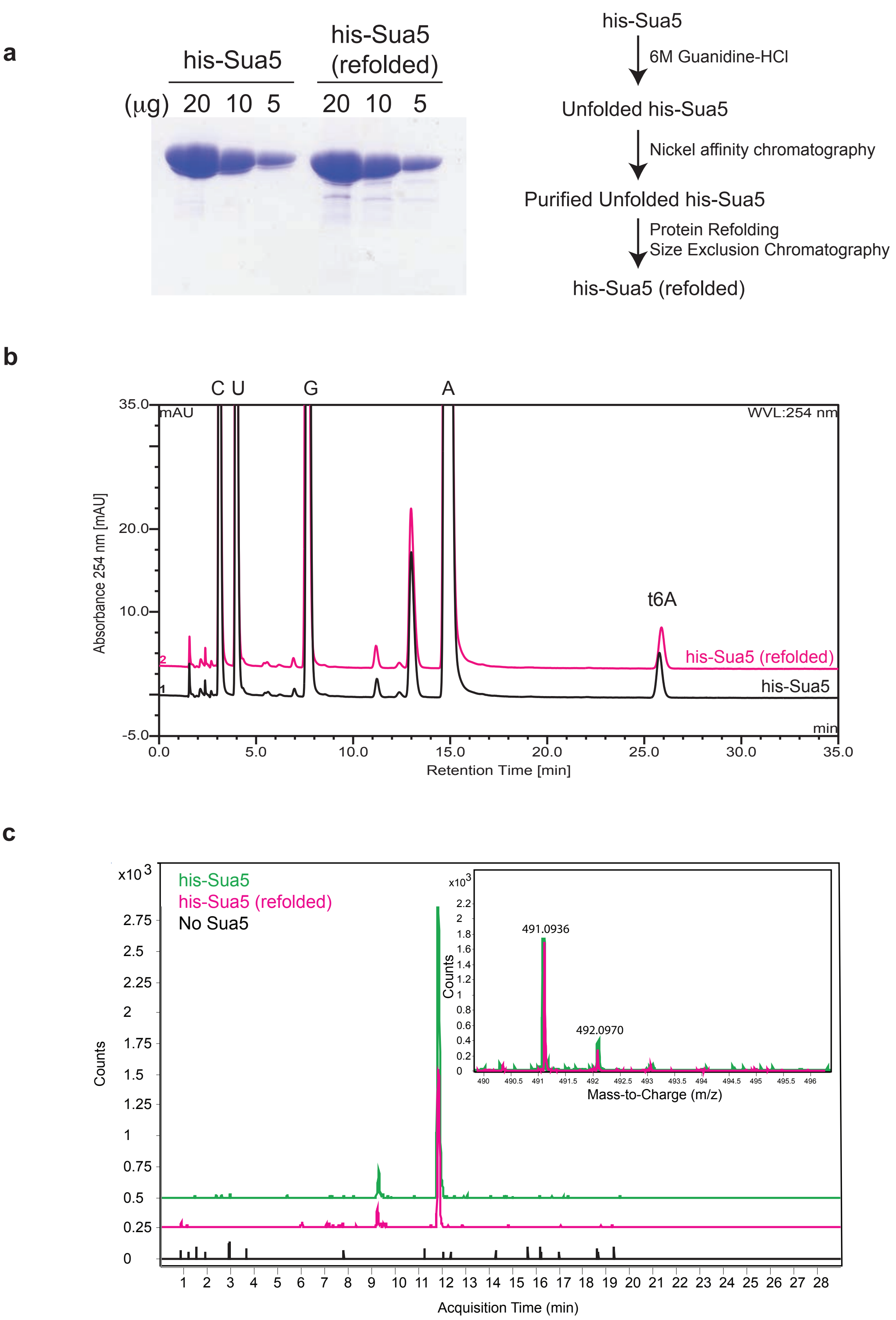

Supplementary Figure 4

a

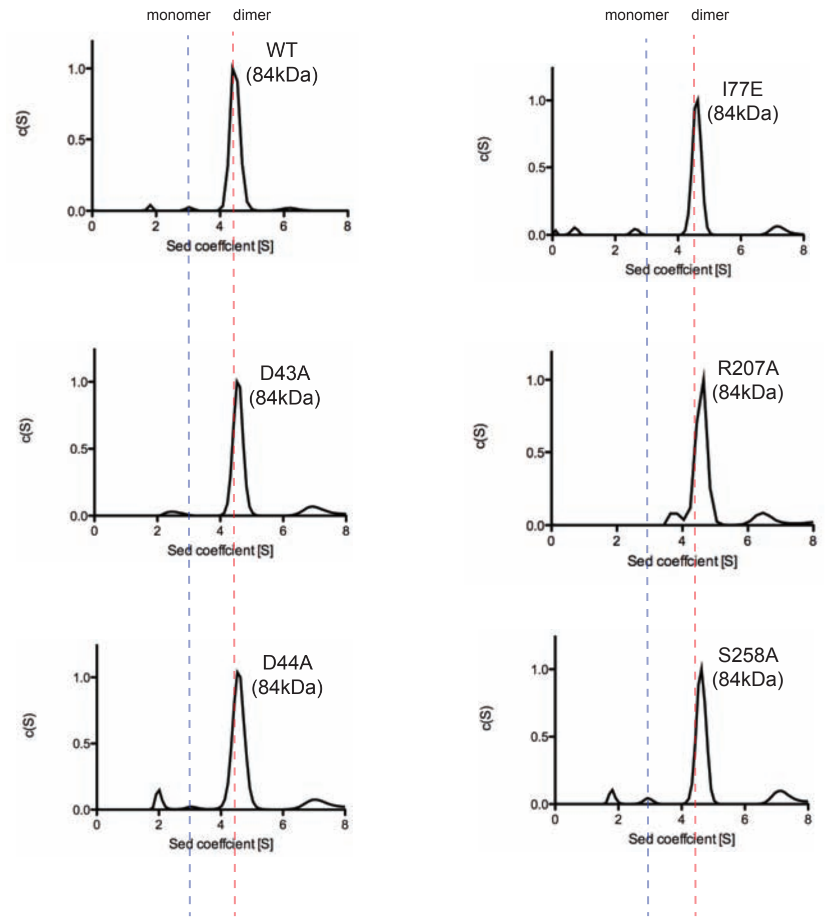

b

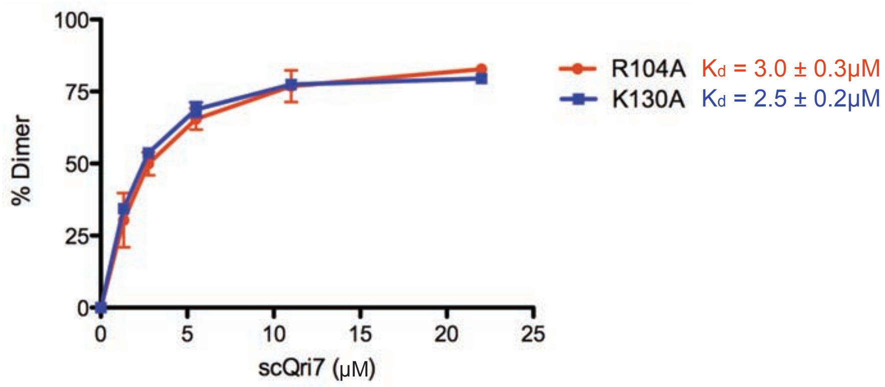

Supplementary Figure 5
